# The Buffalo Obstetrical Society

**Published:** 1891-03

**Authors:** 


The Buffalo Obstetrical Society, at its last annual meeting, elected the following officers for the ensuing year: President, Dr. A. H. Briggs; Vice-President, Dr. DeLancey Rochester; Secretary and Treasurer, Dr. William C. Krauss.

